# Fatal Hypoglycemia in a Nondiabetic Patient With End-Stage Familial Hypertrophic Cardiomyopathy on SGLT2 Inhibitor Therapy

**DOI:** 10.1016/j.jaccas.2026.107191

**Published:** 2026-03-06

**Authors:** Yanqiu Li, Li Zhong

**Affiliations:** Department of Cardiology, The Third Affiliated Hospital of Chongqing Medical University (Fangda Hospital), Chongqing, China

**Keywords:** adverse drug reaction, dapagliflozin, heart failure, hypertrophic cardiomyopathy, hypoglycemia, multiple organ failure

## Abstract

**Background:**

Sodium-glucose cotransporter 2 inhibitors (SGLT2i) are cornerstone therapies for heart failure, but their safety in extreme clinical scenarios warrants careful evaluation.

**Case Summary:**

A 37-year-old woman with end-stage familial hypertrophic cardiomyopathy and MYH7 mutations developed recurrent hypoglycemia during dapagliflozin therapy. Despite comprehensive guideline-directed therapy, progressive malnutrition (body mass index 18.4 kg/m^2^) and hypoglycemic episodes culminated in fatal hyperlactic acidemia and multiple organ failure.

**Discussion:**

This case demonstrates SGLT2i may precipitate life-threatening hypoglycemia in nondiabetic, malnourished patients with advanced heart failure, particularly when superimposed on end-stage hypertrophic cardiomyopathy's metabolic vulnerability.

**Take-Home Messages:**

Enhanced vigilance and regular glucose monitoring are essential when using SGLT2i in high-risk populations. Unexplained hypoglycemia warrants immediate drug discontinuation in vulnerable patients.

## Visual Summary

**Figure:**
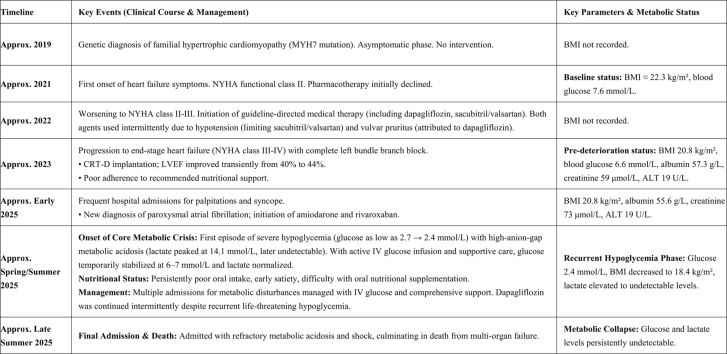

**Visual Summary:**
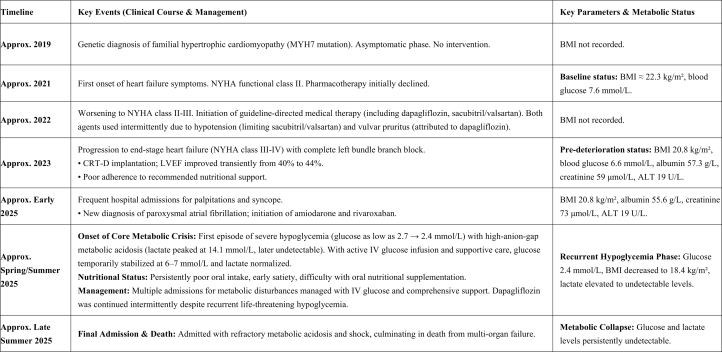
Timeline of Clinical Course, Management, and Metabolic Parameters

## History of Presentation

A 37-year-old woman with a family history of autosomal dominant familial hypertrophic cardiomyopathy (HCM) presented with a multiyear progressive heart failure course. Initially an asymptomatic MYH7 mutation carrier (genetically confirmed in 2019), she developed her first heart failure symptoms (NYHA class II) in 2021. Her condition deteriorated to NYHA class II and III in 2022, leading to the initiation of guideline-directed medical therapy. In mid-2023, she experienced significant decline (NYHA class III-IV) with complete left bundle branch block, necessitating cardiac resynchronization therapy defibrillator (CRT-D) implantation, which temporarily improved her ejection fraction and functional status.

A pivotal shift in her clinical course began in early 2025, characterized by recurrent hospitalizations for palpitations, syncope, and severe metabolic crises. These episodes featured recurrent, profound hypoglycemia and lactic acidosis, consistently linked to periods of poor oral intake. Despite multiple life-threatening hypoglycemic events, dapagliflozin was persistently administered. Her final admission in late Summer 2025 was marked by refractory metabolic acidosis, septic shock, and multiorgan failure, culminating in her death 1 week later.

## Past Medical History

### Familial HCM

Genetically confirmed (MYH7: c.4384G>A, c.1357C>T).

### Strong family history

**Figure 1:**
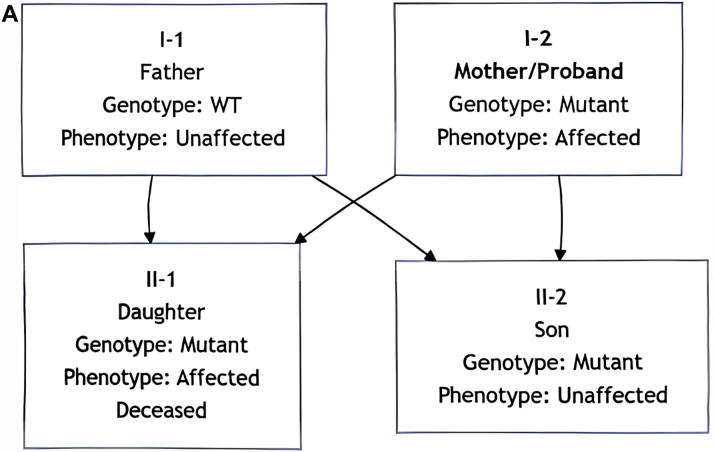

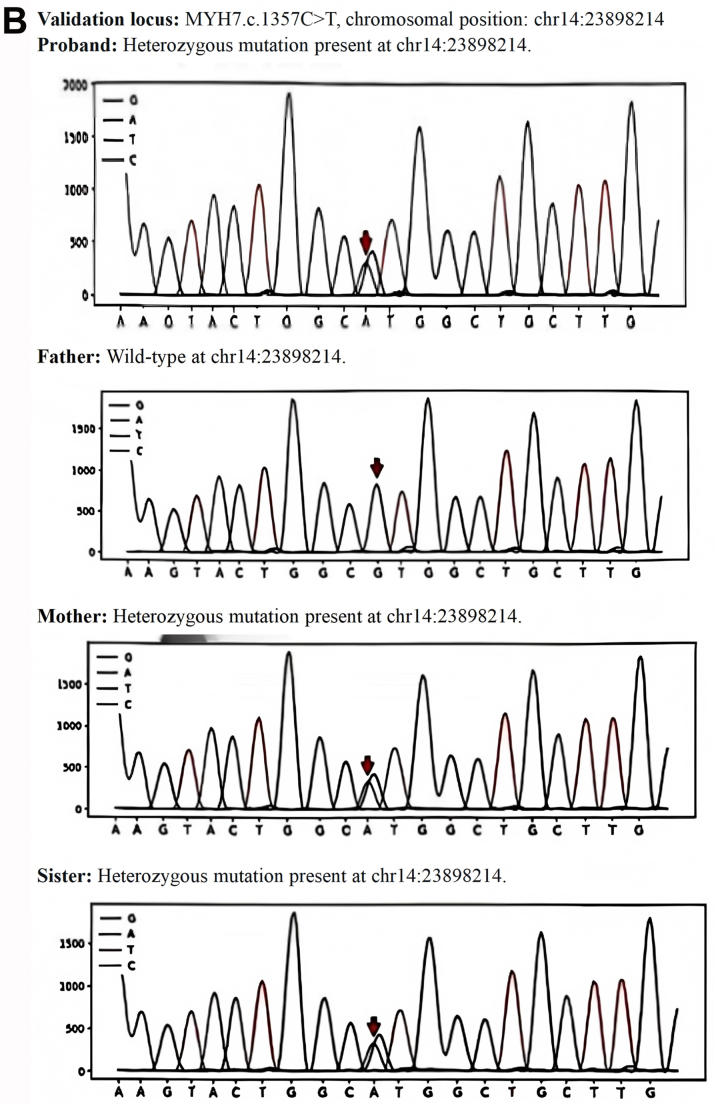

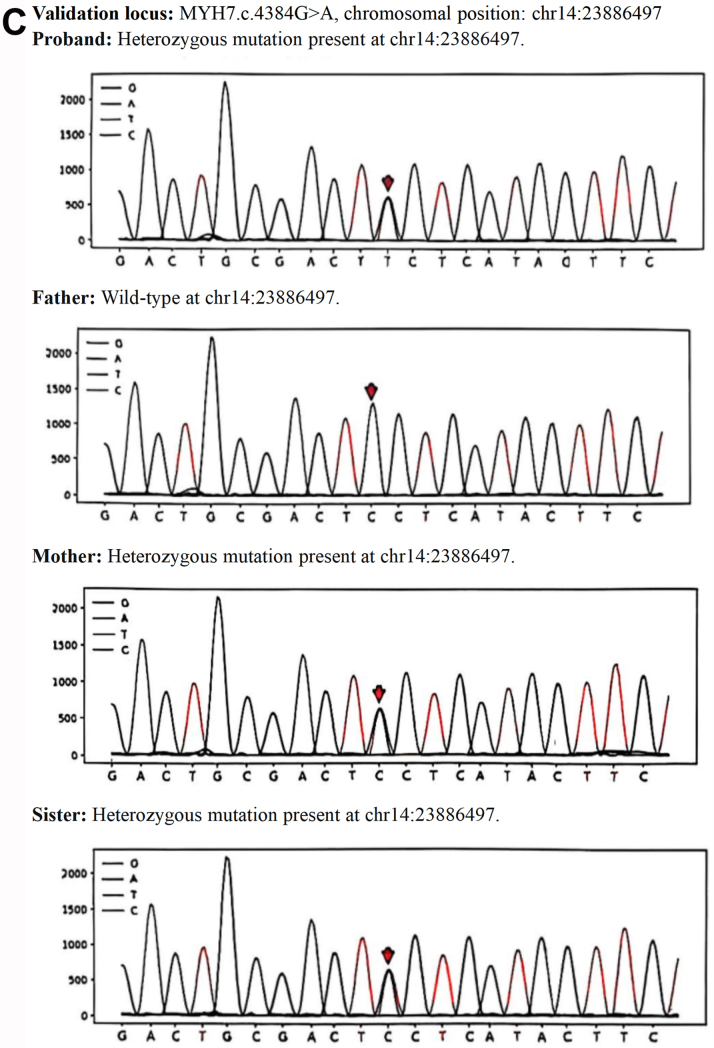
Family Pedigree and Genetic Sequencing (A) Pedigree of the family with autosomal dominant genetic disorder. The arrow indicates the proband (I-2, mother and current case). The slash denotes deceased individuals. Genetic analysis confirmed the presence of the pathogenic mutation in the proband (I-2), deceased daughter (II-1), and the son (II-2). The father (I-1) is wild-type (WT). The son (II-2) is an asymptomatic mutation carrier. (B) MYH7 Gene c.1357C>T locus family validation sequencing chromatogram. (C) MYH7 Gene c.4384G>A locus family validation sequencing chromatogram.

Autosomal dominant inheritance pattern was observed. The parents and spouse did not carry the pathogenic variant. The daughter died of heart failure at age 12 due to this condition, and the son is an asymptomatic carrier (Figure 1A).

### Heart failure

NYHA functional class II (2021) progressing to end-stage (NYHA functional class III and IV) by 2023.

### Cardiac device

CRT-D with left bundle branch area pacing (implanted in mid-2023).

### Arrhythmia

Paroxysmal atrial fibrillation (diagnosed in early 2025).

### Nutritional status

Progressive cachexia was documented, with body mass index (BMI) declining from approximately 22 kg/m^2^ in 2021 to 18.4 kg/m^2^ by mid-2025 (Visual Summary). The patient reported persistent anorexia and early satiety, primarily attributed to congestive symptoms of advanced heart failure and concomitant anxiety. A formal evaluation ruled out anorexia nervosa. Throughout her multiple admissions, nutritional consultations consistently recommended high-calorie oral supplements (eg, protein powder). However, adherence and efficacy were severely limited by poor oral intake and intermittent nausea, constituting a major ongoing clinical challenge.

### Consideration of advanced therapies

Given her end-stage status (NYHA functional class III and IV), the treatment team evaluated advanced options including heart transplantation. CRT-D implantation was performed in 2023 as a bridge therapy. However, heart transplantation was ultimately not pursued due to a combination of factors including limited organ availability and the patient's socioeconomic circumstances.

### Other

Drug-induced hypotension (limiting guideline-directed medical therapy tolerance), vulvar pruritus (attributed to dapagliflozin), and diagnosed anxiety disorder with panic attacks (Spring 2025).

## Differential Diagnosis

1. Sodium-glucose cotransporter 2 inhibitor (SGLT2i)-associated hypoglycemia: the primary concern, given the strong temporal relationship between dapagliflozin use and recurrent hypoglycemic episodes in a nondiabetic, malnourished patient.
2. Sepsis-induced metabolic acidosis and hypoglycemia: considered, especially during the final admission with elevated procalcitonin and white blood cell.
3. Cardiogenic shock with end-organ hypoperfusion: A constant consideration given her underlying end-stage heart failure, which can cause lactic acidosis.
4. Anxiety disorder with panic attacks and hyperventilation: Initially suspected to explain palpitations and syncope, but unable to account for the objective metabolic derangements.
5. Inborn error of metabolism (unmasked in adulthood): a less likely possibility, though the recurrent lactic acidosis prompts consideration.

## Investigations

### Genetic analysis

Confirmed pathogenic MYH7 mutations (c.4384G>A, c.1357C>T) (Figures 1B and 1C).

#### Cardiac imaging and function

##### Echocardiography

Documented an initial improvement in left ventricular ejection fraction from 40% (pre-CRT-D) to 44% (post-CRT-D implantation in 2023), followed by a terminal decline to 30%. Serial studies showed no significant left ventricular outflow tract obstruction.

##### Chest radiography

**Figure 2:**
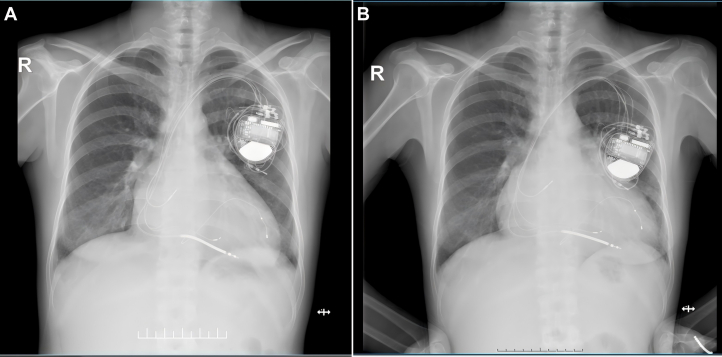
Serial Chest X-ray Imaging (A) Chest x-ray post-cardiac resynchronization therapy procedure. (B) Chest x-ray at 1-year follow-up after CRT implantation.

Demonstrated progressive cardiomegaly (Figures 2A and 2B).

#### Serial laboratory profiles

##### Metabolic and nutritional trends

Longitudinal monitoring revealed progressive decline (Visual Summary). Serial arterial blood gas analyses consistently showed profound hypoglycemia (glucose as low as 2.4 mmol/L) with high-anion gap metabolic acidosis (predominantly lactic acidosis). Nutritional markers were indicative of cachexia, with a BMI of 18.4 kg/m^2^ and fluctuating prealbumin levels suggesting intermittent protein-energy malnutrition.

##### Renal and hepatic function

Blood urea nitrogen showed a rising trend from 2025, correlating with worsening renal function. Liver enzymes remained largely normal until a transient, marked elevation of alanine aminotransferase coincided with the final metabolic crisis in mid-2025.

##### Terminal admission

Key findings included hyperkalemia (K^+^ 8.4 mmol/L), leukocytosis, and markedly elevated procalcitonin (>100 ng/mL).

#### Other diagnostics

##### Electrocardiography and device interrogation

**Figure 3:**
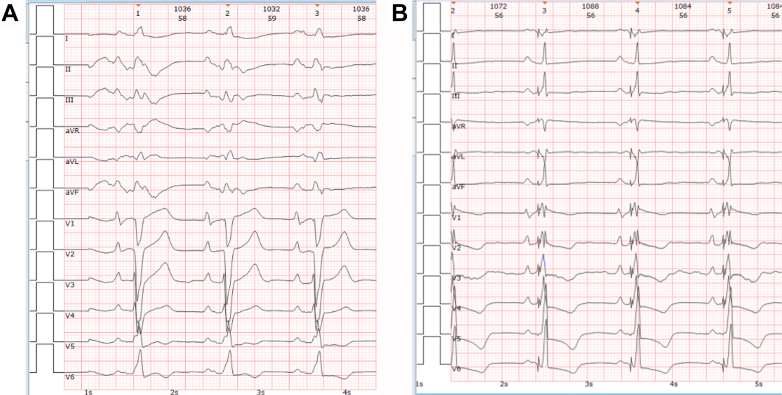
Electrocardiographic Changes Before and After Cardiac Resynchronization Therapy Defibrillator Implantation (A) Preoperative ECG for CRT-D: sinus rhythm at 59 beats/min, complete left bundle branch block, QRS duration of 166 ms, and poor R-wave progression in leads V_1_ to V_3_. (B) Postoperative ECG for CRT-D: sinus rhythm at 67 beats/min, ventricular paced rhythm, with QRS duration of 140 ms. CRT-D = cardiac resynchronization therapy defibrillator; ECG = electrocardiography.

Revealed complete left bundle branch block followed by post-CRT QRS narrowing (Figures 3A and 3B). Device interrogation ruled out significant arrhythmias during later syncopal episodes.

##### Therapy-related assessment

The patient was never treated with a cardiac myosin inhibitor, as there was no echocardiographic evidence of obstruction to warrant its use.

## Management

### Pharmacotherapy

A complex regimen of guideline-directed medical therapy was attempted, including metoprolol, spironolactone, vericiguat, and intermittent sacubitril/valsartan. Amiodarone and rivaroxaban were added for atrial fibrillation. Dapagliflozin was continued intermittently despite recurrent hypoglycemia.

### Device therapy

#### Successful CRT-D implantation with echocardiographic optimization

##### Acute metabolic management

Repeated admissions required intravenous glucose supplementation to correct hypoglycemia and lactic acidosis.

##### Psychiatric care

Initiated fluoxetine, alprazolam, and mirtazapine for anxiety, which were later discontinued due to somnolence.

##### Terminal care

Required mechanical ventilation, continuous renal replacement therapy, vasopressors, and broad-spectrum antibiotics for septic shock.

## Outcome and Follow-Up

The patient's clinical course was marked by progressive metabolic instability and heart failure, with multiple hospitalizations between Spring and mid-Summer 2025 for hypoglycemic crises. Despite transient improvements with supportive care, she was re-admitted in late Summer 2025, in a state of refractory metabolic acidosis and shock. The persistent administration of dapagliflozin, despite clear evidence of its association with life-threatening hypoglycemia, represented a critical oversight. Despite maximal intensive care support, she developed irreversible multiorgan failure and died 1 week after her final admission.

## Discussion

SGLT2i represent a cornerstone therapy in contemporary heart failure management, supported by robust evidence demonstrating reductions in mortality and hospitalization.^1^ However, their safety profile in patients with extreme clinical phenotypes, such as end-stage HCM complicated by cachexia, remains less defined.^2^ This report details a fatal case of recurrent hypoglycemia following dapagliflozin initiation in a nondiabetic woman with end-stage familial HCM, highlighting critical considerations for drug safety in uniquely vulnerable populations.

## Therapeutic Decision-Making in a Nonstandard Indication

The decision to initiate dapagliflozin in this patient was driven by the extrapolation of its compelling benefits in broad heart failure populations to her advanced, refractory clinical state despite HCM not being a formal approved indication.^3^ This reflects a real-world clinical dilemma where the potent mortality benefit of SGLT2i in systolic heart failure prompts their consideration in advanced heart failure of varied etiologies, even in the absence of condition-specific trial data.^4^ Our experience underscores the necessity of a more nuanced application, where the profound phenotypic and genotypic particularities of HCM—namely, an inherent myocardial energy deficit—demand exceptional caution.^5^

## Reevaluating the Role of Hypoglycemia in a Multifactorial Demise

While the immediate cause of death was multiorgan failure secondary to refractory metabolic acidosis and septic shock, recurrent, severe hypoglycemia constituted a critical and preventable metabolic trigger. Substantial daily urinary glucose excretion induced by SGLT2 inhibition, estimated at 60 to 80 g/d, imposed a significant caloric drain.^6^ In the setting of advanced HCM with baseline energetic impairment and superimposed severe cachexia (BMI 18.4 kg/m^2^), this created a perilous synergy. The resulting hypoglycemic crises likely exacerbated myocardial energetic bankruptcy and electrical instability, accelerating the downward clinical spiral. Thus, hypoglycemia was not the sole cause but a pivotal precipitant within a “perfect metabolic storm.”

## Cachexia and Beta-Blockade: Risk Accumulation and Diagnostic Challenge

The role of progressive cachexia in this case cannot be overstated. Malnutrition and low BMI are increasingly recognized as independent risk factors for adverse events, including hypoglycemia, in heart failure patients on SGLT2i therapy.^7^ The patient's profound weight loss signified a catabolic state with depleted glycogen reserves and impaired gluconeogenesis, leaving no metabolic buffer against the glucosuric effect of dapagliflozin. More importantly, concomitant beta-blocker therapy significantly compounded the diagnostic complexity. Beta-blockers suppress the characteristic adrenergic response to hypoglycemia (eg, palpitations, tremor, diaphoresis), thereby masking early warning symptoms, as robustly documented in clinical studies.^8^ In this case, the patient presented only with nonspecific symptoms such as palpitations and chest tightness, initially attributed to heart failure progression—a classic manifestation of this masking effect. This “dual masking”—cachexia weakening metabolic reserve and beta-blockade concealing clinical symptoms—made hypoglycemia exceptionally difficult to detect in a timely manner, constituting a unique challenge in the management of high-risk patients.

## Potential Pharmacogenetic Vulnerabilities

Beyond the primary pathogenic MYH7 mutation, genetic predisposition to adverse drug reactions warrants consideration. Polymorphisms in genes involved in drug metabolism, such as UGT1A9 which mediates dapagliflozin glucuronidation, could theoretically alter drug clearance and increase systemic exposure.^9^ While not investigated here, the occurrence of a severe, recurrent adverse effect in this patient raises a plausible hypothesis for underlying pharmacogenetic susceptibility. This highlights an important area for future research to identify genetic markers that may predict SGLT2i-associated metabolic risks in vulnerable subgroups.^10^

## Conclusion and Clinical Implications

This fatal outcome mandates a paradigm of heightened vigilance. In nondiabetic patients with advanced heart failure—particularly those with energy-deficient conditions like HCM, significant cachexia, and concurrent beta-blocker use—SGLT2i therapy requires extreme caution, meticulous patient selection, and proactive nutritional assessment. It is crucial to recognize that beta-blockers may mask hypoglycemic symptoms; therefore, proactive and regular blood glucose monitoring (rather than reliance on symptoms alone) is essential for patients on this high-risk combination. Any unexplained hypoglycemic event must prompt immediate drug discontinuation. Our experience advocates for a shift from a blanket class-based recommendation to a rigorously individualized risk-benefit framework, prioritizing immediate metabolic safety alongside long-term cardiorenal protection.

## Funding Support and Author Disclosures

This work was supported by the Internal Research Fund of The Third Affiliated Hospital of Chongqing Medical University (grant number: KY24044). The funder had no role in the design, conduct, or reporting of this work. The authors have reported that they have no relationships relevant to the contents of this paper to disclose.

**Take-Home Messages**

• Enhanced vigilance and regular glucose monitoring are essential when using sodium-glucose cotransporter 2 inhibitors in high-risk populations.
• Unexplained hypoglycemia warrants immediate drug discontinuation in vulnerable patients.
